# Autologous hematopoietic stem cell infusion for sustained myelosuppression after BCMA–CAR-T therapy in patient with relapsed myeloma

**DOI:** 10.1038/s41409-019-0674-2

**Published:** 2019-09-19

**Authors:** Quande Lin, Xiyang Liu, Lu Han, Lina Liu, Baijun Fang, Quanli Gao, Yongping Song

**Affiliations:** Henan Cancer Hospital, Affiliated Cancer Hospital of Zhengzhou University, 127 Dongming Road, 450008 Zhengzhou, China

**Keywords:** Stem-cell research, Preclinical research

To the Editor,

Chimeric antigen receptor (CAR) T-cell targeted multiple myeloma antigens such as CD138, kappa-light chain, and B-cell maturation antigen (BCMA) as well as CD19 had been widely adopted. More and more CAR-T clinical trials for MM presented encouraging results [1–3]. However, current CAR-T treatment still faces the adverse reactions as cytokine release syndrome (CRS), myelosuppression, and other complications, especially, severe myelosuppression is often a fatal threat to patients. We adopted the stem cell infusion to promote hematopoietic recovery for a relapsed MM patient developing severe and persistent myelosuppression after CAR-T cell therapy.

**Fig. 1:**
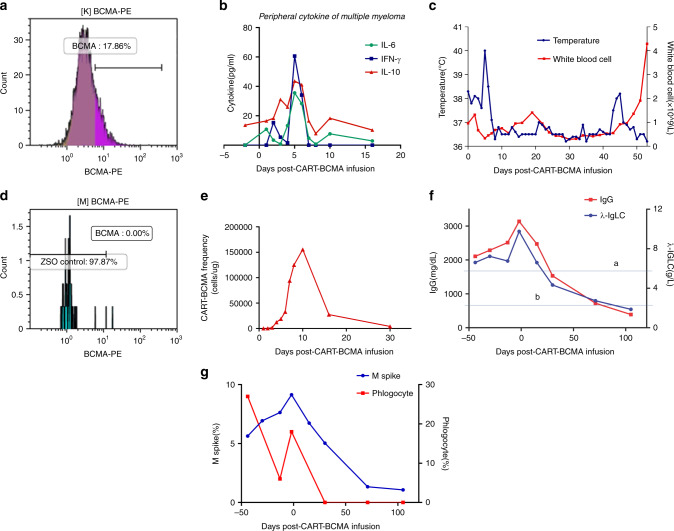
**a** BCMA-positive myeloma cells on 17 days before CAR-T cell therapy detected by flow cytometry. **b** Measures of INF-γ, IL-6, and IL-10 after chimeric antigen receptor (CAR)-T BCMA cells infusion. **c** Changes of temperature and white blood cells after CAR-T BCMA cells infusion (a is the time of BCMA CAR-T cells infusion and b is the time of stem cell infusion). **d** BCMA-positive myeloma cells on 30 days after CAR-T cell therapy detected by flow cytometry. **e** CAR-T cell frequency after BCMA CAR-T cells infusion. **f** Changes of IgG and λ-IgLC before and after BCMA CAR-T cells infusion (The line a is the upper normal limit of IgG (1800 mg/dL) and the line b is the upper normal limit of λ-IgLC (2.42 g/L)). **g** Changes of M spike and phlogocyte in bone marrow before and after BCMA CAR-T cells infusion

A male patient with 56 years old was diagnosed with multiple myeloma, IgG, lambda type, stage II for ISS stage, and III for RISS stage. He had received nine courses of treatment including one course of PD regimen (bortezomib combined with dexamethasone) and eight courses of PAD regimen (bortezomib, epirubicin, and dexamethasone) before auto-stem cell transplantation (ASCT) and achieved the complete remission according to the evaluation criteria of the IMWG. Sufficient number of hematopoietic stem cells (6.2 × 10^8^/kg for MNC and 5.6 × 10^8^/kg for CD34^+^ cells) were harvested. On November 23, 2017, the patient received high-dose melphalan (200 mg/m^2^) and performed ASCT, hematopoiesis recovered 15 days after stem cell infusion. Lenalidomide (25 mg/day × 21 days) combined with dexamethasone (40 mg/week) as maintenance therapy started 1 month after transplantation. However, serum IgG and M spike increased, myeloma cells reached to 12.8% after 3 months of maintenance therapy. He was recruited for BCMA targeted CAR-T clinical trial (NCT03664661) under a consent form approved by the Ethics Counselor of Henan Cancer Hospital since the myeloma cells expressed BCMA antigen detected by flow cytometry (Fig. 1a). Peripheral blood mononuclear cells were collected, autologous T cells were purified and transduced with a lentiviral vector encoding a CAR incorporating an anti-BCMA scFv, 4-1BB costimulatory, and CD3-zeta T-cell activation domain. Patient underwent lymphodepletion with FC regimen (Fludarabine 25 mg/m^2^ daily for 3 days, Cyclophosphamide 500 mg/m^2^ daily for 2 days) and was infused with a total of 1.0 × 10^7^/kg anti-BCMA CAR-T cells on June 16 and 17, respectively.

During the following 5 days after infusion, the patient experienced sustained fever and temperature peaked at 40.2 °C but there was no significant increase in C-reactive protein and serum procalcitonin levels. Patient developed a grade 2 CRS according to Lee’s grading system [4] as serum cytokine levels significantly elevated, including INF-γ, IL-6, and IL-10 (Fig. 1b, c). The temperature dropped to normal and the cytokine level decreased after supportive treatment for 7 days. Patient developed a grade IV of myelosuppression and was treated with granulocyte-stimulating factor, suspended red blood cells, and platelet transfusion as well as other supportive care. On day 30, BCMA expression disappeared while CAR-T cells remained detectable in BM (Fig. 1d, e). However, the patient was still at grade IV of myelosuppression until 45 days after CAR-T cells infusion. In order to promote hematopoietic recovery, autologous stem cells were infused on July 30 and August 2, respectively with a total of MNC 2.10 × 10^7^/kg and CD34^+^ cell 1.80 × 10^6^/kg. The peripheral blood cells recovered 10 days after autologous stem cell infusion (Fig. 1c). The condition improved gradually, and achieved VGPR 105 days after CAR-T cells infusion (Fig. 1f, g).

Previous reports of unexpected organ injury and deaths related by CAR-T cell therapy significantly limit the application of novel therapies [5]. The main adverse effects of CAR-T cell therapy include CRS, neurotoxicity, B cell aplasia, and bone marrow depression [6, 7]. B cell aplasia occurs in almost all patients treated with CAR-T therapy [8]. Myelosuppression is a common but not serious adverse reaction in CAR-T therapy. Cohen reported 11 relapsed and refractory MM patients received BCMA targeted CAR-T cells, anemia, neutropenia, lymphopenia, thrombocytopenia, hypofibrinogenemia occurred in each patient [9]. Fried analyzed the persistent severe hematologic toxicity after CD19 CAR-T cells therapy for patients with relapsed and refractory leukemia and lymphoma [10], it was indicated severe myelosuppression was more common in patients with high grade CRS and in patients receiving stem cell transplantation prior to CAR-T therapy. The patient underwent ASCT within 1 year before receiving the CAR-T cell infusion, insufficient recovery of bone marrow hematopoiesis due to intensive chemotherapy, which in turn may result in bone marrow dysfunction under hematopoietic stress.

Most myelosuppression could be restored with component transfusion and stimulating factor applications [11]. However, the case we presented here obtained mild CRS and no neurotoxicity occurred, but severe and lasting myelosuppression were observed. We found that severe myelosuppression already existed when he received infusion of CAR-T cells (Fig. 1c), this is consistent with the report [10]. Applications of the component transfusion and stimulating factor did not improve the myelosuppression, ultimately, it was successfully recovered by autologous stem cell infusion.

The application of autologous stem cells in the CAR-T therapy, whose effect was similar to the role of hematopoietic stem cell infusion in transplantation after high-dose chemotherapy [12], promoted the reconstruction of bone marrow hematopoietic system, avoided serious infection. Autologous stem cell infusion is also a worthwhile option when patients suffer from severe and sustained myelosuppression after treatment with CAR-T cells.
